# Colorectal Laterally Spreading Tumors by Computed Tomographic Colonography

**DOI:** 10.3390/ijms141223629

**Published:** 2013-12-03

**Authors:** Yasuo Kakugawa, Yutaka Saito, Takahisa Matsuda, Takeshi Nakajima, Mototaka Miyake, Gen Iinuma

**Affiliations:** 1 Division of Screening Technology and Development, Research Center for Cancer Prevention and Screening, National Cancer Center, 5-1-1 Tsukiji, Chuo-ku, Tokyo 104-0045, Japan; E-Mail: giinuma@ncc.go.jp (G.I.); 2 Endoscopy Division, National Cancer Center Hospital, 5-1-1 Tsukiji, Chuo-ku, Tokyo 104-0045, Japan; E-Mails: ysaito@ncc.go.jp (Y.S.); tamatsud@ncc.go.jp (T.M.); tnakajim@ncc.go.jp (T.N.); 3 Diagnostic Radiation Division, National Cancer Center Hospital, 5-1-1 Tsukiji, Chuo-ku, Tokyo 104-0045, Japan; E-Mail: mmiyake@ncc.go.jp

**Keywords:** colonoscopy, colorectal cancer, computed tomographic colonography, laterally spreading tumor (LST)

## Abstract

To date, few reports focused primarily on detecting colorectal laterally spreading tumors (LSTs) have been published. The aim of this study was to determine the visibility of LSTs on computed tomographic colonography (CTC) compared with that on colonoscopy as a standard. We retrospectively reviewed and matched data on endoscopic and CTC reports in 157 patients (161 LSTs) who received a multidetector CT scan using contrast media immediately after total colonoscopy at the National Cancer Center Hospital in Tokyo, Japan, between December 2005 and August 2010. The results of the total colonoscopy were known at the time of the CTC procedure and reading. Of the 161 LSTs detected on colonoscopy, 138 were observed and matched by CTC (86%). Of the 91 granular type LSTs (LST-Gs), 88 (97%) were observed and matched, while of the 70 non-granular type LSTs (LST-NGs), 50 (71%) were observed and matched by CTC (*p* < 0.0001). CTC enabled observation of 73% (22/30) of 20–29 mm, 83% (35/42) of 30–39 mm, 88% (49/56) of 40–59 mm, and 97% (32/33) of ≥60 mm tumors. The rate of observed LSTs by CTC was 86% (97% of LST-G, 71% of LST-NG) of the LSTs found during total colonoscopy.

## 1. Introduction

Colorectal cancer is a major cause of cancer mortality worldwide. Early detection and removal of colorectal neoplasms is the most effective way to minimize cancer mortality [1,2]. Currently, colonoscopy is the gold standard for the investigation of the colorectum, offering a sensitive method for luminal examination.

In contrast, computed tomographic colonography (CTC) is a relatively new radiological technique for imaging the entire colorectum, and it has received widespread attention as an alternative to conventional colonoscopy for colorectal cancer screening [3]. By means of CTC, images can be reconstructed in almost any plane and can be used to create three-dimensional images. CTC is thought to be less invasive than colonoscopy and it requires less manpower [3–5]. The sensitivity of CTC for detecting polyps ≥ 10 mm has been reported to be 90% [4].

It is reported that most flat lesions can be detected by CTC [6–18]; however, the term “flat” seems to be something of a misnomer because the majority of non-polypoid lesions are superficially elevated and are not completely flat or even with the mucosa [18]. In contrast, laterally spreading tumors (LSTs) among the flat lesions are well known among the endoscopists worldwide, and they are clearly defined as lesions of ≥10 mm in diameter with a low vertical axis extending laterally along the interior luminal wall [19,20]. The LSTs are thought to be one of the important precursor lesions of colorectal cancer [21] and, therefore, clinically important to detect in earlier stage.

The aim of this study was to determine the visibility of LSTs on CTC compared with that on colonoscopy as the standard.

## 2. Results

### 2.1. Patient Characteristics

Table 1 shows a summary of 161 LSTs in 157 patients who underwent a multidetector CT scan using contrast media immediately after total colonoscopy at our facility. The median age was 64 years (range, 32–83 years) and the male/female ratio was 1.2 (86/71). There were 91 LSTs granular type (LST-G) and 70 LSTs non-granular type (LST-NG). The median tumor size was 40 mm (range, 20–115 mm). Twenty-six lesions and 135 lesions were histopathologically diagnosed as adenomas and cancers [intramucosal (m) 99; sm_1_, 19; and sm_2_, 17], respectively. Among the 26 adenomas, 12 and 14 lesions were LST-G and LST-NG, respectively. Among the 135 cancers, 79 and 56 lesions were LST-G (m, 67; sm_1_, 4; and sm_2_, 8) and LST-NG (m, 32; sm_1_, 15; and sm_2_, 9), respectively. There were no complications reported during the CTC procedures.

### 2.2. Overall Visibility Rate with CTC

**Figure 1.**
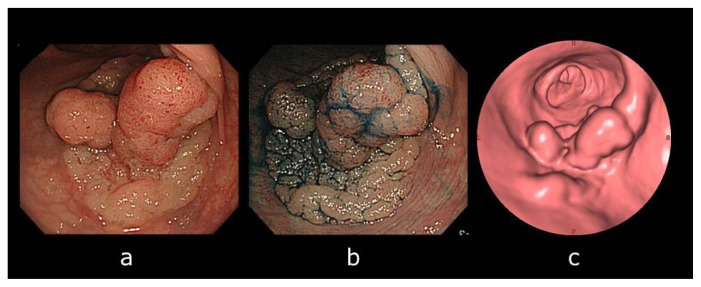
A laterally spreading tumor (LST) observed by computed tomographic colonography (CTC) in a 75-year-old man. (**a**) Colonoscopy revealed a Is + IIa (LST granular type: LST-G), 55 mm in diameter located in the rectum; (**b**) The lesion became apparent after indigo–carmine dye spraying; and (**c**) The surface endoluminal virtual image revealed the lesion on CTC. Endoscopic submucosal dissection (ESD) was subsequently performed on this LST-G lesion. Pathological diagnosis was a tubular adenoma with high- and low-grade dysplasia.

CTC enabled the observation and matching of LSTs in 138 (86%) of the 161 total LSTs, in 16 (62%) of the 26 colorectal adenomas, and in 122 (90%) of the 135 colorectal cancer lesions in this study (Table 2). A representative example of the LSTs observed by CTC is shown in Figure 1.

### 2.3. Visibility Rates According to Factors

Of the 91 LST-Gs, 88 (97%) were observed and matched, while of the 70 LST-NGs, 50 (71%) were observed and matched by CTC (*p* < 0.0001; Table 2). For colorectal adenomas, of the 12 LST-Gs, 10 (83%) were observed and matched. Of the 14 LST-NGs, 6 (43%) were observed and matched by CTC. For colorectal cancers, of the 79 LST-Gs, 78 (99%) were observed and matched. Of the 56 LST-NGs, 44 (79%) were observed and matched by CTC. A significant correlation was seen between the visibility rate and LST invasion depth (*p* = 0.0006). Any visibility rate of LST-Gs was >80% regardless of invasion depth, while the visibility rate of every LST-NGs was lower as compared to LST-Gs, in particular, adenomatous LST-NG was only 43%.

**Figure 2.**
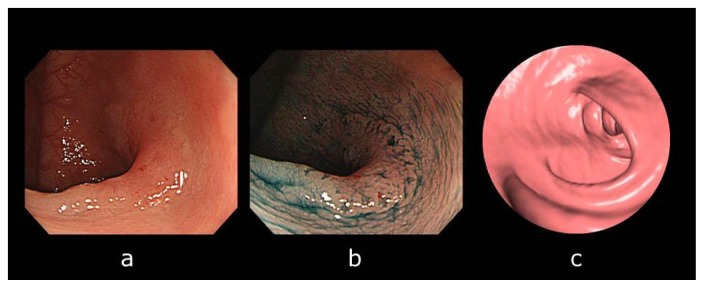
A laterally spreading tumor (LST) not observed by computed tomographic colonography (CTC) in a 44-year-old man. (**a**) Colonoscopy revealed an IIa + IIc (LST non-granular type: LST-NG) lesions, 25 mm in diameter located in the transverse colon; (**b**) The lesion margin became apparent after indigo-carmine dye spraying; and (**c**) We could not observe the lesion by means of CTC at the time of the analysis even when we knew the results of the colonoscopy. Endoscopic submucosal dissection (ESD) was subsequently performed on this LST-NG lesion. Pathological diagnosis was a well-differentiated adenocarcinoma with an sm_2_ invasion depth of 1500 μm from the musclaris mucosae. Because the invasion depth was sm_2_, an additional surgery was performed.

Lesion-matching results suggest that overall 14% (23/161) of lesions could not be observed by CTC. Of the 23 overlooked lesions, 20 (87%) were LST-NGs, defined as lacking an elevated component. Representative examples of LSTs overlooked by CTC are shown in Figure 2. No elevated component was observed even in the colonoscopy view.

CTC resulted in the observation rate of 73% (22/30) of 20–29 mm, 83% (35/42) of 30–39 mm, 88% (49/56) of 40–59 mm, and 97% (32/33) of ≥60 mm tumors (Table 3). The visibility rate of LSTs increased significantly with the lesion size (*p* = 0.0416).

With regard to the tumor location, CTC resulted in the observation rate of 100% (17/17) lesions in the cecum, 74% (43/58) in the right colon, 79% (31/39) in the left colon, and 100% (47/47) in the rectum (Table 4). The visibility rate in the cecum and rectum was, therefore, 100% (64/64), whereas that in the remaining colon (the right and left colon) was 76% (74/97; *p* < 0.0001).

## 3. Discussion

This is the first study reported about LST visibility on CTC from the viewpoint of Japanese endoscopists. LSTs which had been also called as “carpet lesion” are considered to be one of the important precursor lesions of colorectal cancer [21]; in particular, LST-NGs have a higher potential for malignancy [22]. Therefore, endoscopists usually observe the colorectal mucosa carefully in order to detect such lesions during a colonoscopic procedure, but the literature suggests that it is somewhat difficult to detect such lesions even with high-resolution colonoscopy [23]. Using CTC, which is a relatively new radiological modality, we clarified the visibility rate of LSTs compared with that colonoscopy as the standard.

It has been reported that most flat lesions can be detected by CTC [6–17]. The term flat, however, seems to be a misnomer because the majority of non-polypoid lesions are superficially elevated and are not completely flat [18]. Thus, we focused on LSTs, which are clearly defined as lesions of ≥10 mm in diameter with a low vertical axis extending laterally along the interior luminal wall [19,20].

Recently, growing evidence supports the theory that superficial submucosal (sm) invasive cancers <1000 μm (sm_1_) without unfavorable histology do not involve lymph node metastasis [24]. Such lesions can be treated endoscopically; therefore, the need for surgery can be avoided, resulting in a better quality of life for patients. Any neoplastic lesion including LSTs should be detected at an early stage when the lesion’s depth is superficial and should be resected by the subsequently performed colonoscopy. In this study, therefore, we focused on patients with LSTs suitable for ESD without the necessity of surgery.

Our study clarified that most LST-Gs could be observed by CTC; however, LST-NGs could not be observed so much. The visibility rate of LST-NG was 71% even using contrast media. Unless we had used contrast media, the visibility rate would have been even lower. In this study, of the 23 overlooked lesions, 20 (87%) were LST-NGs. This result suggests that the most important factor of overlooking a lesion during CTC examination is the lack of the elevated component in the lesion. LST-NG usually has only a faint redness; therefore, it is comparably difficult to detect them even using high-resolution colonoscopy. It is, therefore, impossible to detect such a color change using CTC. Our results are different from the study result by Pickhardt *et al.* [18]. They reported that among 9152 consecutive individuals undergoing initial CTC, 18 colorectal carpet neoplasms in 18 patients were identified and were neoplastic lesions. They stated that most neoplastic lesions in the form of carpet lesions can be detected by CTC. In contrast, our data showed that some LSTs could not be observed by CTC, even though they are adenomatous lesions. Lesion-matching results suggest that overall 14% (23/161) of lesions could not be observed by CTC. We believe there could be three reasons for this discrepancy.

First, the preceding examination was different between the two studies. We performed colonoscopy first, and then if LSTs were detected, we performed CTC. Pickhardt *et al*. performed CTC first, then if carpet lesions were detected, confirmed them by colonoscopy. There may be some overlooked flat neoplastic lesions on CTC because of the absence of colonoscopic confirmation in subjects without any colorectal lesions. Second, the Japanese colonoscopic technique and bowel preparation are remarkably advanced [25]; therefore, in this study, the rate of detection of LSTs on colonoscopy, which was assumed to be the gold standard, could be high. Therefore, the observation rate of LSTs on CTC could be decreased. Third, the definitions of terms carpet lesion and LST may be different. The number of LST-NG might be lower in the study by Pickhardt *et al.* [18].

The visibility rate for lesions in the right and left colon was lower than that in the cecum and rectum. Although the reason for such a difference is unknown, this finding could be important for optimization of CTC. Our study suggests that CTC is a safe method because there were no procedure-related complications. Another advantage of CTC is that it is not necessary to sedate the patient during the examination, and the lymph nodes, liver, and the lungs, which are frequent sites of colorectal cancer metastasis, can be inspected by performing CT screening of the entire body. In Japan, CTC has been gradually accepted in clinical practice. The cost of CTC and total colonoscopy without endoscopic treatment is approximately 30,000 and 20,000 yen, respectively. In this study, we did not calculate the time required for performing those procedures, but in our experience, each of them should be completed within 15 min.

There are several limitations in this retrospective study. First, only patients with endoscopically diagnosed LSTs were included. Second, the participating radiologists knew the results of the colonoscopic examination before performing and evaluating each CTC. Third, the CTCs were clinically performed using contrast media. These factors could lead to a higher visibility rate than expected in a daily practice. A prospective double-blind study is warranted to further clarify the detectability of LSTs when using CTC without contrast media in an asymptomatic population.

In conclusion, the rate of observed LSTs when using CTC was 86% (97% of LST-G, 71% of LST-NG) of the LSTs found during total colonoscopy, with a wide variation depending on macroscopic type, size, and invasion depth.

## 4. Materials and Methods

### 4.1. Study Patients

We retrospectively reviewed data from 157 patients with 161 LSTs who received a contrast-enhanced multidetector computed tomography (MDCT) scan immediately after total colonoscopy for the pretreatment assessment of endoscopic submucosal dissection (ESD) between December 2005 and August 2010, at the National Cancer Center Hospital in Tokyo, Japan.

### 4.2. Endoscopic Procedure

In accordance with standard colonoscopic preparations in Japan, all patients received 2 or 3 L of polyethylene glycol electrolyte solution for colonic preparation on the day of examination. An anti-peristaltic agent (10 mg of scopolamine butylbromide or 0.5 mg of glucagon) was injected intravenously as a mandatory course, while a sedative (2–3 mg of midazolam) was injected intravenously only when necessary.

If a lesion was detected during a colonoscopic procedure, the surface was washed with a lukewarm solution containing pronase. The lesion was then examined in detail in the conventional views, and pictures of the lesion were obtained. In addition, 0.4% indigo-carmine dye was sprayed on the lesion to enhance its surface detail so that a high magnification observation could be performed to evaluate the surface texture (pit pattern) to differentiate an invasive pattern from a non-invasive pattern [26,27].

The LST is defined as a lesion of ≥10 mm in diameter with a low vertical axis extending laterally along the interior luminal wall [19,20]. LSTs are further classified on the basis of their macroscopic appearance. The LST-G is defined by the presence of aggregates of even or uneven nodules on the surface, whereas the LST-NG has a smooth surface lacking granular formations [19,20]. Tumor size was determined endoscopically and the tumors were also categorized into 4 groups according to their size: 20–29 mm, 30–39 mm, 40–59 mm, and ≥60 mm. Each lesion was identified as being located in the cecum, right colon (ascending and transverse colon), left colon (descending and sigmoid colon), or rectum. After completion of the total colonoscopy, we suctioned any remaining liquid as thoroughly as possible.

### 4.3. CTC Procedure

If an eligible LST was identified during the total colonoscopy as possibly being suitable for ESD, informed written consent was obtained from the patient. The patient underwent MDCT using a contrast medium immediately without stool tagging after the total colonoscopy.

Before the CTC procedure, an intramuscular injection of an antiperistaltic agent (20 mg of scopolamine butylbromide) was administered and a rectal tube was inserted and then gently insufflated with carbon dioxide to the maximum level tolerated by the patient. Initially, the CTC was performed with the patient in a supine position and it was subsequently repeated with the patient in a prone position.

CTC was performed on a 64× multidetector CT scanner (Aquilion, Toshiba Medical Systems, Tokyo, Japan). Scans were obtained through the abdomen and pelvis by using the following parameters: 120 kV; 200–400 mA with automatic exposure control; 64 rows × 0.5 mm collimation; and helical pitch, 53 (pitch factor 0.828). Each patient received an intravenous bolus injection of 150 mL of a contrast medium, namely, iohexol 350 (Omnipaque, Daiichi-Sankyo Pharmaceutical, Tokyo, Japan), from a power injector at the rate of 3 mL/s through a 20-gauge plastic IV catheter placed in an antecubital vein. The entire abdomen was scanned during the arterial phase 50 s after introduction of the contrast material. All images were reconstructed at a thickness of 1 mm and the slices were transferred to an image workstation (Ziostation, Zio, Tokyo, Japan) to generate 3D images for each patient.

Two radiologists (MM, GI) specializing in the gastrointestinal tract analyzed the CTC findings. Each radiologist had experience with more than 500 CTC examinations before the beginning of this study. Usually, the 2-dimensional CT was read first, followed by the 3-dimensional CT. The information in the colonoscopy report and photographs showing the presence and location of any LSTs detected were freely available at the time of the CTC procedure.

### 4.4. Pathological Examination

An ESD procedure was subsequently performed within 2–3 weeks on any detected LST if there were no invasive endoscopic findings, lymph-node metastasis, or distant metastasis revealed by CT examination. All resected specimens were immediately fixed in a 10% buffered formalin solution and stained with hematoxylin-eosin. Tissue specimens were examined by expert pathologists with histopathological diagnoses based on the Vienna classification [28]. According to the Paris classification [24], lesions with a vertical invasion depth <1000 μm in the submucosal layer were classified as sm superficial and lesions with an invasion depth ≥1000 μm were considered as submucosal deep invasive cancers (sm-deep).

### 4.5. Statistical Analysis

The significance of proportional differences was assessed by Fisher’s exact test and SPSS statistical software (SPSS for Windows, version 16.0J, Tokyo, Japan). Statistical significance was defined as *p* < 0.05.

## 5. Conclusions

The rate of observed LSTs using CTC was 86% (97% of LST-G, 71% of LST-NG) of the LSTs found during total colonoscopy, with a wide variation depending on macroscopic type, size, and invasion depth.

## Figures and Tables

**Table 1.** Patient characteristics.

**Age**	Median (Range)	64 (32–83) years old
**Gender**	Male/Female	86/71
**Macroscopic Type**	LST-G/LST-NG	91/70 lesions
**Location**	Cecum/Right Colon/Left Colon/Rectum	17/58/39/47 lesions
**Size**	Median (Range)	40 (20–115) mm
**Histopathological Diagnosis**	Adenoma	26 lesions
	Cancer	135 lesions
	m	99 lesions
	sm_1_	19 lesions
	sm_2_ or deeper	17 lesions

LST-G, laterally spreading tumor granular type; LST-NG, laterally spreading tumor non-granular type; m, intramucosal cancer; sm_1_, submucosal cancer <1000 μm; sm_2_ or deeper, submucosal cancer ≥1000 μm.

**Table 2.**
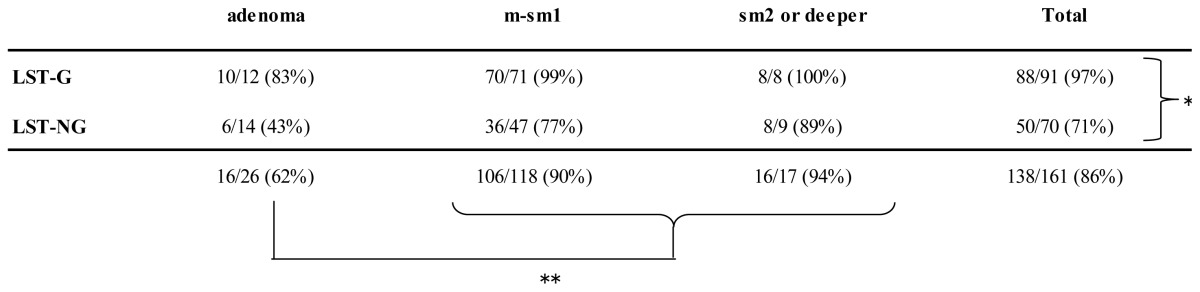
Visibility rate by tumor invasion depth.

* A significant correlation was observed between the visibility rate of LST-G and LST-NG (*p* < 0.0001);

** A significant correlation was observed between the visibility rate and LST invasion depth (*p* = 0.0006); LST-G, laterally spreading tumor granular type; LST-NG, laterally spreading tumor non-granular type; m, intramucosal cancer; sm_1_, submucosal cancer <1000 μm; sm_2_ or deeper, submucosal cancer ≥1000 μm.

**Table 3.**
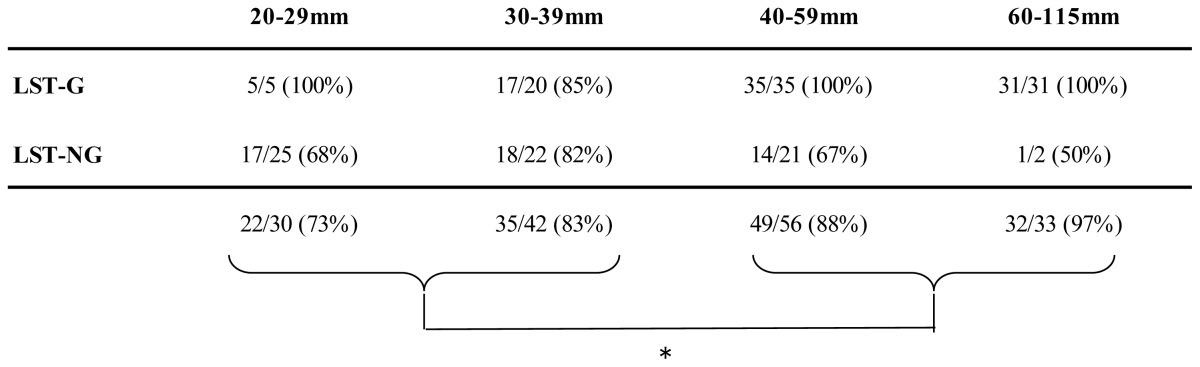
Visibility rate by macroscopic type and lesion size.

* Visibility rate of LSTs significantly increased with the lesion size (*p* = 0.0416); LST-G, Laterally Spreading Tumor Granular Type; LST-NG, Laterally Spreading Tumor Non-granular Type.

**Table 4.** Visibility rate according to LST location.

	Cecum	Right Colon	Left Colon	Rectum
**LST-G**	15/15 (100%)	20/22 (91%)	12/13 (92%)	41/41 (100%)
**LST-NG**	2/2 (100%)	23/36 (64%)	19/26 (73%)	6/6 (100%)

	17/17 (100%)	43/58 (74%)	31/39 (79%)	47/47 (100%)

LST-G, laterally spreading tumor granular type; LST-NG, laterally spreading tumor non-granular type.
